# Supplementary material for: Identification of photoperiod-induced specific miRNAs in the adrenal glands of Sunite sheep (Ovis aries)
Source: Front Vet Sci. 2022 Jul 22;9:888207. doi: 10.3389/fvets.2022.888207 (PMC9354845; doi:10.3389/fvets.2022.888207)
Supplement: Supplementary file 1 [file Data_Sheet_1.ZIP › Supplementary file/miRDeep2 file.zip!/miRDeep2 file/Know_Structure/LP42A2/oar-mir-10a.pdf]

miRBase precursor : oar-mir-10a
Total read count : 135622
oar-miR-10a read count : 134804
remaining reads : 818

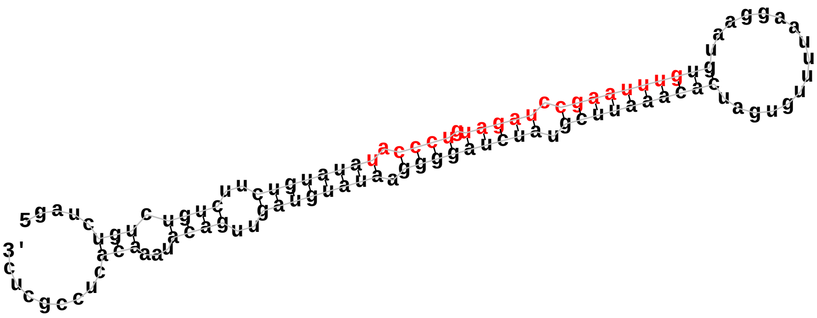

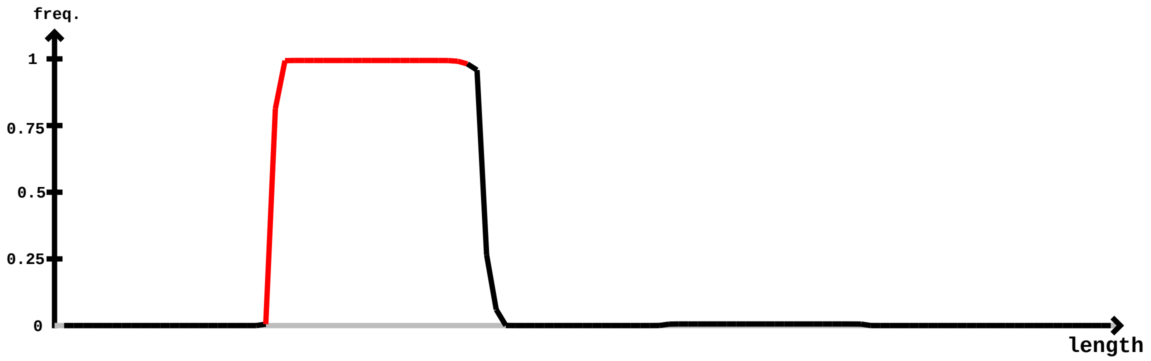

oar-miR-10a

5'- gaucugucugucuucuguauauacccuguagauccgaauuuguguaaggaauuuugugaucacaaauucguaucuaggggaauauguaguugacauaaacacuccgcuc -3'	exp		
....(((.((((..((((((((.((((.(((((.((((((((((...............)))))))))).))))))))).)))))))).))))...)))).......	reads	mm	sample
.aucugucugucuucuguaua..	1	0	seq
..ucugucugucuucuguaua..	1	0	seq
...cugucugucuucuguaua..	1	0	seq
....................auacccuguagauccga..	1	0	seq
....................auacccuguagauccgaauu...	3	0	seq
....................auacccuguagauccgaauuu..	38	0	seq
....................auacccuguagauccgaauuug...	105	0	seq
....................auacccuguagauccgaauuugu..	520	0	seq
....................auacccuguagauccgaauuugug...	35	0	seq
....................auacccuguagauccgaauuugugu..	4	0	seq
.....................uacccuguagauccgaa...	4	0	seq
.....................uacccuguagauccgaau..	33	0	seq
.....................uacccuguagauccgaauu...	290	0	seq
.....................uacccuguagauccgaauuu..	1131	0	seq
.....................uacccuguagauccgaauuug...	2640	0	seq
.....................uacccuguagauccgaauuugu..	82004	0	seq
.....................uacccuguagauccgaauuugug...	18994	0	seq
.....................uacccuguagauccgaauuugugu..	4478	0	seq
.....................uacccuguagauccgaauuugugua...	22	0	seq
.....................uacccuguagauccgaauuuguguaa..	1	0	seq
......................acccuguagauccgaau..	3	0	seq
......................acccuguagauccgaauu...	47	0	seq
......................acccuguagauccgaauuu..	194	0	seq
......................acccuguagauccgaauuug...	390	0	seq
......................acccuguagauccgaauuugu..	11418	0	seq
......................acccuguagauccgaauuugug...	8762	0	seq
......................acccuguagauccgaauuugugu..	3595	0	seq
......................acccuguagauccgaauuugugua...	16	0	seq
.......................cccuguagauccgaauu...	2	0	seq
.......................cccuguagauccgaauuug...	2	0	seq
.......................cccuguagauccgaauuugu..	16	0	seq
.......................cccuguagauccgaauuugug...	36	0	seq
.......................cccuguagauccgaauuugugu..	17	0	seq
.......................cccuguagauccgaauuugugua...	2	0	seq
........................ccuguagauccgaauuugug...	1	0	seq

oar-miR-10a

gaucugucugucuucuguauauacccuguagauccgaauuuguguaaggaauuuugugaucacaaauucguaucuaggggaauauguaguugacauaaacacuccgcuc			
....uguaaggaauuuugugaucaca...	1	0	seq
....ucacaaauucguaucuagggga............................	3	0	seq
....cacaaauucguaucuaggggaa...........................	2	0	seq
....acaaauucguaucuagggga............................	1	0	seq
....acaaauucguaucuaggggaa...........................	3	0	seq
....acaaauucguaucuaggggaau..........................	25	0	seq
....caaauucguaucuagggga............................	9	0	seq
....caaauucguaucuaggggaa...........................	32	0	seq
....caaauucguaucuaggggaau..........................	647	0	seq
....caaauucguaucuaggggaaua.........................	3	0	seq
....caaauucguaucuaggggaauau........................	3	0	seq
....aaauucguaucuaggggaau..........................	77	0	seq
....aaauucguaucuaggggaaua.........................	6	0	seq
....aaauucguaucuaggggaauau........................	1	0	seq
....aauucguaucuaggggaauau........................	2	0	seq
